# The Bee as a Model to Investigate Brain and Behavioural Asymmetries

**DOI:** 10.3390/insects5010120

**Published:** 2014-01-02

**Authors:** Elisa Frasnelli, Albrecht Haase, Elisa Rigosi, Gianfranco Anfora, Lesley J. Rogers, Giorgio Vallortigara

**Affiliations:** 1 Center for Mind/Brain Sciences, University of Trento, Corso Bettini 31, I-38068 Rovereto, Italy; E-Mails: albrecht.haase@unitn.it (A.H.); elisa.rigosi@gmail.com (E.R.); giorgio.vallortigara@unitn.it (G.V.); 2 Department of Physics, University of Trento, via Sommarive 14, 38123 Povo, Italy; 3 BIOtech Research Center, Department of Industrial Engineering, via delle Regole 101, 38123 Trento, Italy; 4 IASMA Research and Innovation Center, Fondazione E. Mach, via E. Mach, 1, 38010 S.Michele a/A (TN), Italy; E-Mail: gianfranco.anfora@fmach.it; 5 Centre for Neuroscience and Animal Behaviour, University of New England, Armidale, NSW 2450, Australia; E-Mail: lrogers@une.edu.au

**Keywords:** behavioural asymmetry, lateralization, learning, memory recall, PER, electroantennography, sensilla, 2-photon microscopy, population-level, sociality

## Abstract

The honeybee *Apis mellifera*, with a brain of only 960,000 neurons and the ability to perform sophisticated cognitive tasks, has become an excellent model in life sciences and in particular in cognitive neurosciences. It has been used in our laboratories to investigate brain and behavioural asymmetries, *i.e.*, the different functional specializations of the right and the left sides of the brain. It is well known that bees can learn to associate an odour stimulus with a sugar reward, as demonstrated by extension of the proboscis when presented with the trained odour in the so-called Proboscis Extension Reflex (PER) paradigm. Bees recall this association better when trained using their right antenna than they do when using their left antenna. They also retrieve short-term memory of this task better when using the right antenna. On the other hand, when tested for long-term memory recall, bees respond better when using their left antenna. Here we review a series of behavioural studies investigating bees’ lateralization, integrated with electrophysiological measurements to study asymmetries of olfactory sensitivity, and discuss the possible evolutionary origins of these asymmetries. We also present morphological data obtained by scanning electron microscopy and two-photon microscopy. Finally, a behavioural study conducted in a social context is summarised, showing that honeybees control context-appropriate social interactions using their right antenna, rather than the left, thus suggesting that lateral biases in behaviour might be associated with requirements of social life.

## 1. Introduction

The honeybee *Apis mellifera* L. (Hymenoptera: Apidae), with a brain of only 960,000 neurons [1] and the ability to perform sophisticated cognitive tasks (e.g., [2,3,4,5]), has become an excellent model in life sciences and in particular in cognitive neurosciences. This insect has been used in our laboratories to investigate brain and behavioural asymmetries using a number of different methods [6]. We are particularly interested in the different functional specializations of the right and the left sides of the brain [7].

Until some three decades ago brain lateralization had been considered to be unique to the human species but then it was discovered to be present in birds and rodents (summarised in [8]). Now we know that it is a feature of the vertebrate brain beginning with the earliest vertebrates [9,10,11] and furthermore evidence is accumulating of its presence in invertebrates also [12]. Since we do not know whether lateralization in vertebrates and invertebrates is homologous or analogous, at this stage it is most valuable to study it in invertebrates in their own right. The honeybee has emerged as a model species to do just this. Moreover, comparison of honeybees and other bee species, particularly those that exhibit primitive social behaviour or that are not social, is informative in testing the hypothesis that directional biases in behaviour, and related morphology, evolved in association with sociality (explained below). To date most of the investigation of laterality in bees has involved olfaction, and only one study has reported lateralization of visual responses. The findings will be summarised with a view to pointing out that bees provide an excellent means of studying the behavioural, structural and molecular aspects of laterality.

## 2. Behavioural Studies (Summarized in Table 1)

**Figure 1:**
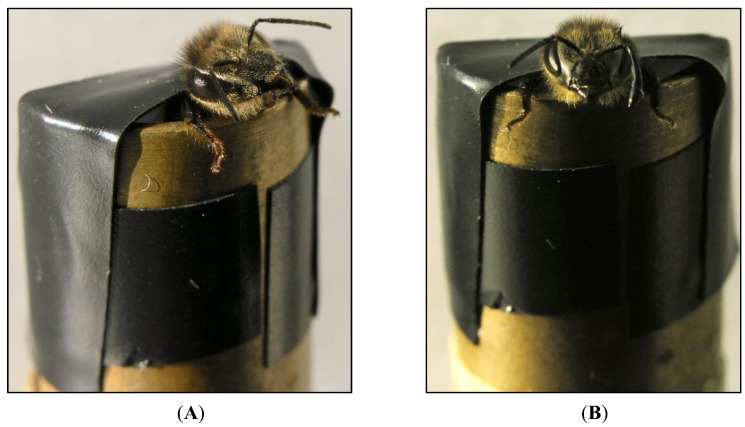
Proboscis Extension Reflex (PER) paradigm. (**A**) Harnessed bee with both antennae in use. (**B**) Harnessed bee with only the right antenna in use—the left antenna is coated with a black silicone compound. (Photo by Elisa Rigosi).

The Proboscis Extension Reflex (PER) paradigm [13] is a well-known classical conditioning paradigm widely used with honeybees. Restrained bees (Figure 1A) are conditioned to extend their proboscis in anticipation of a food (sugar) reward when they perceive an odour stimulus. The first study that reported evidence of lateralization of olfaction in the honeybee *A. mellifera* was conducted by Letzkus and colleagues [14], who investigated asymmetries in olfactory learning performance using two different versions of the PER paradigm. In the first version, honeybees were conditioned to extend their proboscis to a scented drop of sugar water but not to an unscented drop of salt water; in the second version, honeybees were conditioned to extend their proboscis to one odour (lemon essence dissolved in a sugar solution—reward) but not to another odour (vanilla essence dissolved in a salt solution—punishment). Each version of the learning task was carried out on three groups of bees: the bees in one group had their left antenna covered with a silicone compound (Figure 1B), which prevents detection of odour, those in the second group had their right antenna covered, and those in the third group constituted a control in which both antennae were uncovered (Figure 1A). Results of tests, performed the morning after the training and with any covering of antennae still in place, revealed that the bees with the right antenna covered had learnt less well than the bees with their left antenna covered and bees with both antenna uncovered. In fact, the bees trained with only their right antenna in use performed just as well as the untreated controls, suggesting the existence of a population-level lateralization in olfactory learning with a right antennal advantage. The study suggests that bees with only their left antenna in use were incapable of learning and thus of recalling the task using this antenna. However, since the test used was one requiring the bee to recall memory of the task on the day after training and not of learning *per se*, there is another explanation of the results: viz., bees with use of their left antenna only could learn but not recall the task (discussed further below). 

**Table 1:** Summary of the behavioural studies reported in Section 2.

Bee Species	Lateralized Behaviour	References
*Apis mellifera*	Right-antenna advantage in olfactory performance	[14]
	Right-eye advantage in visual performance	[15]
	Use of the right antenna in short-term memory recall and of the left antenna in long-term memory recall: a functional lateral shift	[16]
	Response competition associated with right–left antennal asymmetries of new and old olfactory memory traces	[17]
	Short-term memory lateralization may be odour-specific, or the lateral shift in the transition from short-term to long-term memory occurs at different time scales for different types of odours	[18]
	Right-antenna in social interactions	[7]
*Trigona carbonaria Trigona hockingsi Austroplebeia australis*	Use of the right antenna in short-term memory recall and of the left antenna in long-term memory recall	[19]
*Osmia cornuta*	No right-antenna advantage in olfactory performance	[20]
*Bombus terrestris*	Right-antenna advantage in olfactory performance	[21]
*Bombus* **spp.**	Asymmetry in circling around a vertical inflorescence	[22]
	Social learning drives handedness in nectar-robbing bumblebees	[23]

The PER paradigm was also used by Letzkus *et al.* [15] to compare visual learning in honeybees using their left or right eye. Four groups of bees, all with their antennae removed to facilitate the conditioning to visual stimuli [24], were tested: bees with both eyes covered (BEC), bees with both eyes exposed (BEE), bees with their right eye exposed (REE) and bees with their left eye exposed (LEE). Bees were conditioned to extend their proboscis in anticipation of a food reward according to the colour of a large yellow rectangle presented on a computer-controlled display. Each experiment consisted of two 10-training sessions; the first one was conducted the morning after the eyes had been covered and the second one was conducted the following morning. BEC bees showed no learning throughout the entire training, whereas the BEE bees’ performance rose steadily. REE bees also showed an increase in learning performance, but the response rate was slightly (but not significantly) lower than that of BEE bees throughout the training, reaching the same performance level of BEE at the end of the test. On the contrary, LEE bees reached a mean learning performance significantly lower than that of BEE and REE groups. As found for the olfactory task [14], the results suggested that bees have population-level lateralization of a right-eye advantage in visual performance. However, since the significant difference between the REE and LEE groups did not appear until trials 16 to 20 (*i.e.*, on the final day of testing), it is unclear whether the lateralization was associated primarily with visual learning or with retrieval of long-term memory of the task. This point is relevant as we will explain in discussing the differential roles of the left and right antennae in recall of short- and long-term memory.

Rogers and Vallortigara [16] investigated recall of olfactory memory at various times after honeybees had been trained using both antennae. After PER training with both antennae in use, using lemon plus sucrose solution as the positive stimulus and vanilla plus saturated saline as the negative stimulus, bees were tested for recall 1–2 and 23–24 h later and with the left or right antenna coated with the silicone compound (Figure 1B). At 1–2 h after training, bees showed excellent recall when tested using their right antenna, but poor or no recall when tested using their left antenna, suggesting that short-term memory (STM) is accessed mainly via the right antenna. By contrast, 23–24 h after training recall was good when the left antenna was in use but not when the right antenna was in use, demonstrating that long-term memory (LTM) is accessed mainly via the left antenna. Thus, retrieval of olfactory learning is a time-dependent process.

**Figure 2:**
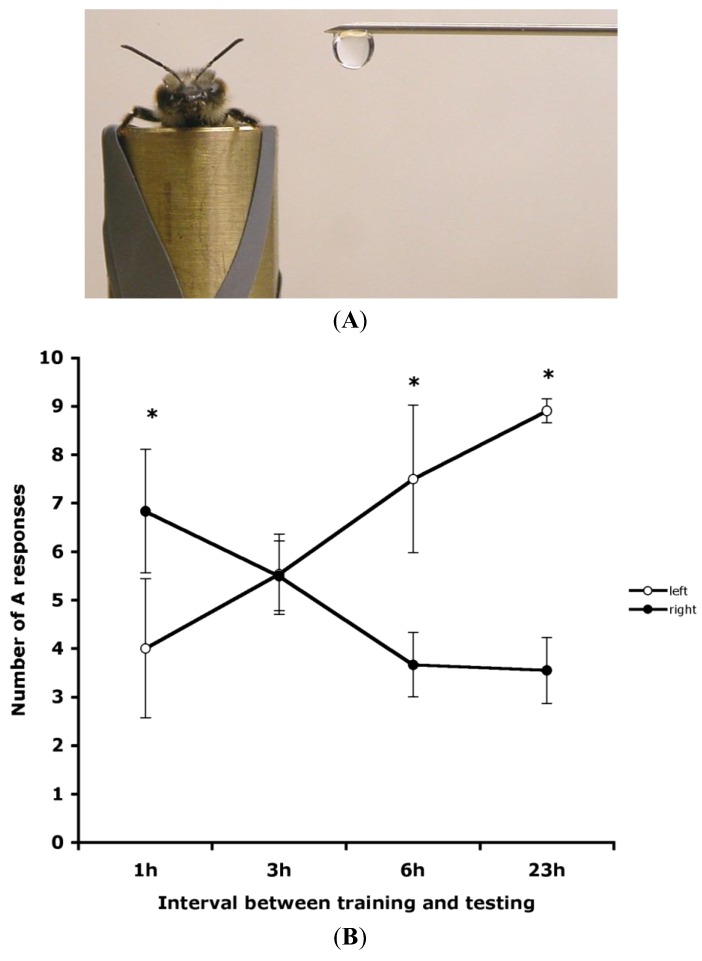
Recall of olfactory memory in honeybees at various times by lateral presentation of the odour. (**A**) A photograph of lateral presentation of the odour. (**B**) The mean number of A responses (*i.e.*, extension of the proboscis to the positive stimulus and no extension to the negative stimulus), plotted with standard error bars, in recall tested at various intervals after training is shown for presentations of the odours on the bee’s left side (open circles) or right side (closed circles). (From [16]).

In a series of experiments on the laterality of response to a conditioned stimulus (CS), *i.e.*, odour stimuli, Sandoz and Menzel [25] showed that honeybees could be trained to produce side-specific responses. Bees conditioned to two different odorants, one being learned on each side, produced rather nonspecific response patterns, responding to both odorants on both sides. When conditioned to an odour on one side only, bees responded after a retention period of three hours to this odour on both sides, suggesting that the learned information is indeed transferred between sides. However, Sandoz and Menzel [25] did not show any asymmetry in the recall of memory. This is maybe due to the retention period of three hours that Sandoz and Menzel [25] used in their experiments. In fact, as Rogers and Vallortigara [16] showed, time is a fundamental factor in the recall of olfactory memories and, as a consequence, also in the antennal asymmetries connected with these processes (see on Figure 2B that the 3 h point is the cross-over time when no laterality is apparent).

Sandoz *et al.* [26] also investigated the side-specificity of the unconditioned stimulus (US), *i.e*., sucrose solution stimulation of the antennae (which triggers the PER) and of the proboscis (where bees lick the sucrose solution). Anatomically, the brain receives unilateral US input through each antenna, but bilateral input from the proboscis. By controlling each US component, Sandoz *et al.* [26] investigated whether bees can learn a CS when associated with any US components, and what effect such learning has on the opposite brain side. They observed that the antenna-US induces both unilateral and bilateral reinforcement processes, whereas the proboscis-US produces only bilateral effects. In particular, in one of the experiments conducted, bees were subjected to two conditioning phases, the side of CS input changing from one phase to the other. For both antenna-US and proboscis-US, after this change of CS input, a drop in responses was observed, indicating that the CS-US association was not yet retrievable on the other brain side. Interestingly, the main difference between the two studies by Sandoz and colleagues [25,26] was the amount of time after which transfer was tested. Sandoz *et al.* [26] tested transfer after a 9-min inter-trial interval, whereas Sandoz and Menzel [25] tested for bilateral transfer at 3 h and 24 h after conditioning. This indicates once more that time is a critical factor for the occurrence of bilateral transfer, suggesting that the first phases of the odour CS-US association are unilateral, but later phases are bilaterally accessible.

To make a comparison between the study by Rogers and Vallortigara [16] and the previous findings, it is important to emphasise the different experimental procedures. All the bees in the study performed by Rogers and Vallortigara [16] were trained (learning phase) using both antennae and only after the training (before the recall test) the left or the right antenna was coated with the silicone compound; whereas the bees tested by Letzkus *et al.* [14] used only one antenna in both the learning and testing phases, and so also did bees tested by Anfora *et al.* [20] to be discussed later. Given that Letzkus and colleagues [14] tested the bees on the day after the training phase, the differences they revealed to some extent confounded learning with long-term memory recall. In their experiment, bees forced to use only their right antenna both learnt and recalled the task a day later. This contrasts the findings of Rogers and Vallortigara [16], in which the bees were trained using both antennae. In this case recall of the memory a day later is possible only when the left antenna is in use. From this, we conclude that the apparent shift from right to left antenna in recall of short- and long-term memory depends on using both antennae during learning. This, of course, is the natural condition.

Rogers and Vallortigara [16] also checked whether the laterality was manifested as side biases to odours presented to the left or right side of the bee without any covering of the antennae, and hence in a more natural condition than in the paradigm requiring an antenna to be coated with silicon compound (Figure 1B). Bees were trained using both antennae and recall was tested at several intervals (1, 3, 6 or 23 h) after training (Figure 2A). The tests used lateral presentation of the positive and negative stimuli and no coating of the antennae. There were significantly more correct PER responses to odours presented to the bee’s right than to the left side at 1 h after training; no significant left/right difference occurred at 3 h after training; at both 6 and 23 h after training PER responses were higher on the left side than on the right side (Figure 2B). These results confirm the time-dependency of the retrieval of olfactory memory, showing that 3 h after training is a transitional period and that short-term memory may be accessed mainly when using the right antenna and long-term memory when using the left antenna.

A similar pattern of lateralized recall of short-term and long-term memory has been shown in three species of Australian stingless bees: *Trigona carbonaria*, *Trigona hockingsi* and *Austroplebeia australis* [19]. Bees were trained using both antennae in the PER paradigm to discriminate lemon plus sucrose solution as the positive stimulus and vanilla plus saturated saline as the negative stimulus. Recall of the olfactory memory at 1 h after training was better when the odour was presented to the right than to the left side of the bees. In contrast, recall at 5 h after training was better when the odour was presented to the left than to the right side of the bees.

Frasnelli *et al.* [17] extended the study by Vallortigara and Rogers [16] by testing lateralized recall of olfactory memory in honeybees at 1 or 6 h after training using different odours. After training with lemon(+)/vanilla(−) or cineol(+)/eugenol(−) recall at 1 h was better when the odour was presented to the right side of the bee than when it was presented to the left side. In contrast, recall at 6 h was better when the odour was presented to the left than to the right side. However, when trained with either a familiar appetitive odour (rose) as a negative stimulus, or with a naturally aversive odour (isoamyl acetate, IAA—alarm pheromone) as a positive stimulus, bees showed suppression of the response to odours presented on both the right and the left sides at 1 h after training (likely due to retroactive inhibition—see [27]) and at 6 h after training proboscis protrusion occurred to both odours on both sides. A possible explanation for this behaviour is that at 6 h, when access to memory has shifted to the left antenna, memory of these familiar odours in the left side of the brain would be present as two mutually exclusive forms. As a result of very long-term memory either biologically encoded or acquired before testing, the memory would be present as rose positive and IAA negative and, as a result of the long-term memory of training, it would be present as rose negative and IAA positive. This would produce response competition. As a direct test of this hypothesis, bees were first trained with unfamiliar lemon(+)/vanilla(−) and then (16 h later) re-trained with vanilla(+)/lemon(−); as predicted, 6 h after re-training proboscis protrusion occurred to both odours on both the left and right side.

The apparent shift in memory from the left to the right side (*i.e.*, STM encoded on right-side and LTM encoded on left-side) could be explained by encoding of the memory in different time frames (short- and long-term) on each side of the brain rather than it being transferred from one side to the other [17]. Hence there would be asymmetry in the effectiveness of recall at different times. Overall, these results [17] confirm the novel finding [16] that in bees there is a difference between the neural circuits accessed via the right antenna and those accessed via the left antenna in retrieving the learnt task. Specifically, bees learn to associate a new odour of a positive stimulus and recall the memory soon after training using neural circuits accessed via their right antenna and that, after a period of a few hours, memory consolidation occurs accompanied by antennal asymmetry, bees now being able to recall the odour mainly when using their left antenna. Moreover, this particular dynamic of memory traces has marked consequences when odours are already known to the bees (either for a biological reason or as a result of previous experience) and are thus already present in the long-term memory store. As a result, response competition arising from multiple memory traces can be observed, with bees showing an unexpected lack of specificity of their longer-term olfactory memories.

A similar behaviour towards an odour with a putative innate biological meaning (linalool, a plant odour) has been observed in bumblebees [28]. Bumblebees performed well in a discriminative task when linalool was the rewarded positive odour and phenylacetaldehyde the unrewarded negative odour, but they had difficulties in learning phenylacetaldehyde as the rewarded positive odour and the linalool as the unrewarded negative odour. As in the case of rose odour, in the honeybees of Frasnelli *et al.* [17], the use of linalool as negative stimulus appeared to disrupt the learning of the alternative odour, possibly due to an innate biological meaning of linalool as a positive stimulus.

A strong effect of the odour used in the PER task on the lateralization of short-term memory recall has been reported in honeybees [18]. A series of behavioural experiments assessed response asymmetry of odour recall following proboscis extension reflex conditioning at 1 h after training using three different plant odours (1-octanol, 2-octanone and (−)-linalool). The training and the following test were performed on three groups of bees (the bees in the first group had both antennae in use—Figure 1A, the bees belonging to the second and the third groups had respectively only the right—Figure 1B—or the left antenna in use during training and recall). After training with 1-octanol and 2-octanone, bees showed no differences in the recall test regardless of whether they had use of only their right antenna, only their left antenna or both antennae. In contrast, bees trained with (−)-linalool showed a significant effect of the antenna in use: bees trained (and tested) with their right antenna in use (Figure 1B) performed significantly better than those with only their left antenna in use and they performed as well as bees with both antennae in use (Figure 1A—[18]). Therefore, in the recall test at 1 h after conditioning, different types of plant odour volatiles manifest asymmetries or not, depending on the biological relevance of the plant compound. Linalool is one of the most common derivates of floral scents playing a crucial role as cue for pollinators [29], whereas 1-octanol and 2-octanone are unspecific and ubiquitous volatiles released from the green organs of the plants and thus of minor importance in pollinator plant interaction. These results [18] suggest that short-term memory lateralization in bees may be odour-specific, or that lateral shift [16] associated with the transition from short-term memory to long-term memory occurs at different time scales for different types of odours. Honeybees can learn complex odour mixtures by using a subset of key odours, such as (−)-linalool [30] and, after conditioning bees to a mixture of odours, it was found that (−)-linalool elicited higher levels of responding than did other components of the mixture presented singly [31]. Since, as this research demonstrates, bees are selective in their responses to odours, the different biological relevance of the odour compounds used by Rigosi *et al.* [18] might be a reason for the observed difference in lateralization.

Hammer *et al.* [32] showed that the PER paradigm may be used with honeybees not only to investigate olfactory learning but also thermal learning. They showed that honeybees can learn to associate a nectar reward with a heated stimulus applied to the antenna to mimic natural contact with a warm flower or nectar-offering forager. Hammer *et al.* [32] looked for possible asymmetric use of the antennae in the thermal learning by comparing two groups of honeybees. In the left group they applied the thermal stimulus only to the left antenna, whereas in the right group they applied the thermal stimulus only to the right antenna. Bees were trained to associate the thermal stimulus with a food reward over 24 trials: 12 CS+ and 12 CS− (unrewarded conditioned stimulus) trials. Four different inter-training intervals (ITIs) were used on different groups: 30, 105, 180 or 255 s. Hammer *et al.* [32] found no overall lateralization. However, at the shortest ITI (= 30 s) performance with the right antenna was significantly better than with the left antenna. This may mean that the right antenna has an advantage over the left even if it is slight. Learning on both sides improves as the ITI gets longer and the left antenna catches up [32].

All the studies presented here deal with the asymmetrical use of the antennae of bees in processes such as olfactory learning and recall of the olfactory short- and long-term memory. However, from a behavioural point of view, until very recently, there had been no evidence about the importance of the asymmetrical use of the antennae in a social context. The social context of lateralized behaviour is of particular interest because it may explain—at least partially—the alignment of lateralization at the population-level in eusocial honeybees [33]. Very recently, in fact, Rogers *et al.* [7] investigated whether the rich social life of honeybees may be linked with population-level biases in antennal use. The authors analysed different aspects of social behaviour (latency to contact, numbers of proboscis extension (PER), number of C-responses, number of mandibulations) in pairs of bees coming from either the same colony or from different colonies and having only their right antenna (left antennae removed) or only their left antenna (right antennae removed) or both antennae intact. A directional bias in the use of antennae for three measures of social interaction: latency, PER and C-responses, was observed. Dyads of bees tested with only their right antennae in use contacted after shorter latency and were significantly more likely to interact positively (proboscis extension) than were dyads of bees with only their left antennae in use. Instead, the latter were more likely to interact negatively (C-responses) even though they were from the same colony. Furthermore, in dyads from different hives C-responses were higher in dyads of bees using only their right antennae than in dyads of bees using only their left antennae. The right antenna seems, therefore, to be specialized not only for learning about new odours associated with food sources but also for exchange of odoriferous information between worker bees and in the control of appropriate social responses. When using the right antenna same-colony bees were also motivated to approach and contact each other. Although the bees with only their left antenna in use did not completely avoid each other, their social behaviour was not context-appropriate, possibly due to an inability to distinguish between hive mates and bees from another hive. Hence, there is evidence that the right antenna is used in social behaviour and this supports the hypothesis that lateral biases in behaviour are associated with requirements of social life.

The alignment of lateralization at the population-level has been suggested to be the result of social pressure and to arise as an evolutionary stable strategy when individual asymmetrical organisms coordinate their behaviour with other asymmetrical organisms (for more details see [33]. Theoretical models based on this hypothesis [34,35] proposed that lateralization at the population-level is more likely to have evolved in social species, whereas lateralization at the individual- but not population-level is more likely to be present in solitary species.

To test this hypothesis in another way, Anfora *et al.* [20,21] investigated whether the olfactory asymmetry found in the eusocial honeybee was a characteristic of other related species of Hymenoptera showing different levels of sociality. They replicated the study by Letzkus *et al.* [14] using the first version of the PER paradigm on honeybees and applied it to another two species of Hymenoptera: mason bees (*Osmia cornuta* (Latreille), Hymenoptera, Megachilidae, Megachilinae, Osmini), a solitary species, and bumblebees (*Bombus terrestris* L., Hymenoptera, Apidae, Apinae, Bombini), an annual primitive eusocial species. In honeybees, recall of the olfactory memory at 1 h after training to associate an odour (β-citronellol) with a sugar reward was better in animals trained and tested using their right than it was when they were using their left antenna, confirming previous findings by Letzkus *et al.* [14] and Rogers and Vallortigara [16]. However, no asymmetry was observed in mason bees [20]. In bumblebees, those with the right antenna in use performed as well as those with both antennae in use, whereas bumblebees with the left antenna in use performed significantly less well [21].

Kells and Goulson [22] reported that bumblebees *Bombus* spp*.* show preferred directions of circling as they visit florets arranged in circles around a vertical inflorescence. In three (*Bombus lapidarius*, *Bombus lucorum and Bombus pascuorum*) out of four species examined, the majority of bumblebees circled in the same direction: two species circled anticlockwise and one clockwise. Interestingly, the researchers did not observe any lateralization in *B. terrestris*. Bumblebees observe and copy the behaviour of others with regard to floral choices [36] and, moreover, they can learn to make nectar-robbing holes in flowers as a result of encountering them [37]. Goulson and colleagues [23] investigated handedness in nectar-robbing bumblebees (*Bombus wurflenii* and *Bombus lucorum*) feeding on *Rhinanthus minor*, a flower that can be robbed from either the right-hand side or the left-hand side and they looked at a possible effect of social learning on handedness. From the analysis of numerous patches of *R. minor* spread across an alpine landscape, the authors observed that each patch was robbed on either the right or the left. The intensity of side bias increased through the season and was strongest in the most heavily robbed patches. Bees within patches seemed to learn robbing strategies (including handedness) from one another, either by direct observation or from experience with the location of holes, leading to rapid frequency-dependent selection for a common strategy, *i.e.*, adopting the same handedness within particular flower patches.

## 3. Electrophysiological and Molecular Studies

In this section we present the studies that shed some light on the electrophysiological and molecular correlates of the behavioural asymmetries discussed above.

**Figure 3:**
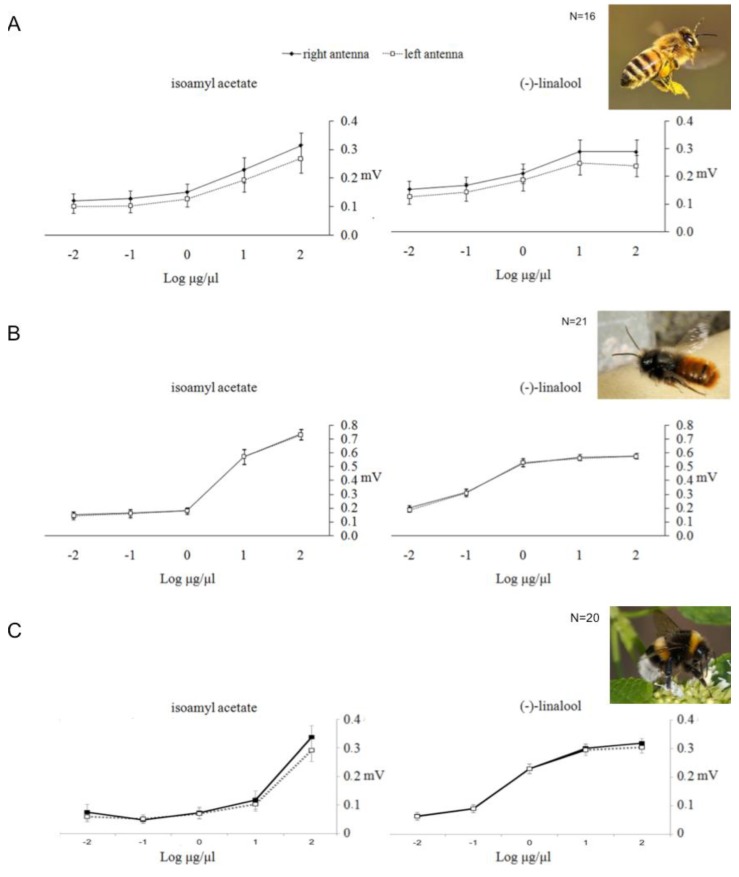
Electroantennographic (EAG) responses. Mean EAG ± SEM absolute responses (mV) of right (unbroken lines with black squares) and left (dotted lines with empty squares) antenna of (**A**) *A. mellifera* (N = 16), (**B**) *O. cornuta* (N = 21) and (**C**) *B. terrestris* foragers (N = 20) to isoamyl acetate (left) and (−)-linalool (right) at five different doses (Log_10_ µg/µL). (From [20,21]).

Anfora *et al.* [20] recorded the electroantennographic (EAG) responses (*i.e.*, a measure of the electrical signal over a section of the antenna, given by the sum of the depolarization potentials of the olfactory receptor neurons in the antenna’s section—[38]) of the right and left antennae of honeybees and mason bees stimulated with two different scents at five different concentrations. In a sample of 16 honeybees, the mean EAG responses to both (−)-linalool and isoamyl acetate (an alarm pheromone) were higher in the right than in the left antenna (mean difference of 18% for the floral volatile compound and 20% for the alarm pheromone), suggesting a population-level lateralization towards the right antenna (Figure 3A). The sample of 21 mason bees did not show, as a group, EAG asymmetry (Figure 3B). However, mason bees appeared to be lateralized at the individual level, in the sense that different individuals showed consistently stronger EAG responses in either the left or the right antenna. In fact, only a minority of individuals showed symmetrical responses, and the large majority, 15 out of 21 individual mason bees, showed significantly stronger responses either with the right (7 individuals) or the left (8 individuals) antenna. In a further study, Anfora and colleagues [21] recorded the EAG responses also on bumblebees using the same odours and concentrations they had used for honeybees and mason bees [20]. No significant differences were observed in the mean EAG responses elicited by either compound between the left and right antennae of the 20 bumblebees analysed (Figure 3C). However, as for mason bees, 12 out of 20 individual bumblebees showed significantly stronger responses either with the right or the left antenna. In mason bees 7 animals showed significant stronger EAG responses with the right antenna and 8 animals with the left antenna, whereas in bumblebees 9 animals showed significant stronger EAG responses with the right antenna and 3 animals with the left antenna.

Biswas and colleagues [39] conducted a further level of analysis of honeybees’ antennal asymmetry by examining the putative “synaptic” role of neuroligins and NrxI in the observed olfactory learning asymmetry. Three groups of bees were assessed: control bees with both antennae intact, bees with only the right antenna (left antenna amputated), and bees with only the left antenna (right antenna amputated). For each group, bees were collected at 24 h, 7 days and 14 days post-emergence. RNA analysis was performed on the dissected brains of the bees at each developmental time point. Data showed that at 24 h post-emergence, bees with only their right antenna showed a slight decrease in levels of neuroligin-1 expression (a protein involved in learning and memory) compared to control bees, which had both antennae intact, whereas bees with only their left antenna had a significantly lower expression level of neuroligin-1. At 7 days, the control bees and bees with only their right antenna intact maintained an equivalent level of neuroligin-1 expression, while bees with only their left antenna showed a higher expression than the other two groups. At 14 days, bees with only their left antenna and the control bees showed similar neuroligin-1 expression, whereas bees with only their right antenna showed a significant increase in neuroligin-1 expression. Broadly speaking, the results by Biswas *et al.* [39] support previous behavioural evidence that learning using the right antenna alone is equivalent to learning using both antennae and that learning performance is significantly poorer when bees use only the left antenna (discussed above).

## 4. Morphological Studies

We have mentioned that the olfactory learning asymmetry in honeybees may be partially due to different sensitivity to odours of the right and the left antenna [20] and to a different level of neuroligin-1 expression [39]. The difference in electroantennographic responses found between the right and left antenna may also be due to a different number of the olfactory receptor neurons in the antenna.

Letzkus *et al.* [14] compared the number of the main olfactory receptor sensory organs in honeybees, *sensilla placodea*, on the two antennae. Images of ten right antennae and ten left antennae (seven of these left-right pairs from the same individuals) were obtained using scanning electron microscopy (SEM) and the mean numbers of *sensilla placodea* per flagellum on the two antennae were compared. The number was significantly higher on the right than on the left antenna (mean difference of 10%). However, only one type of sensilla was considered, and SEM images did not cover the whole antennal segment surface, thus leaving a hidden, non-characterized area.

**Figure 4:**
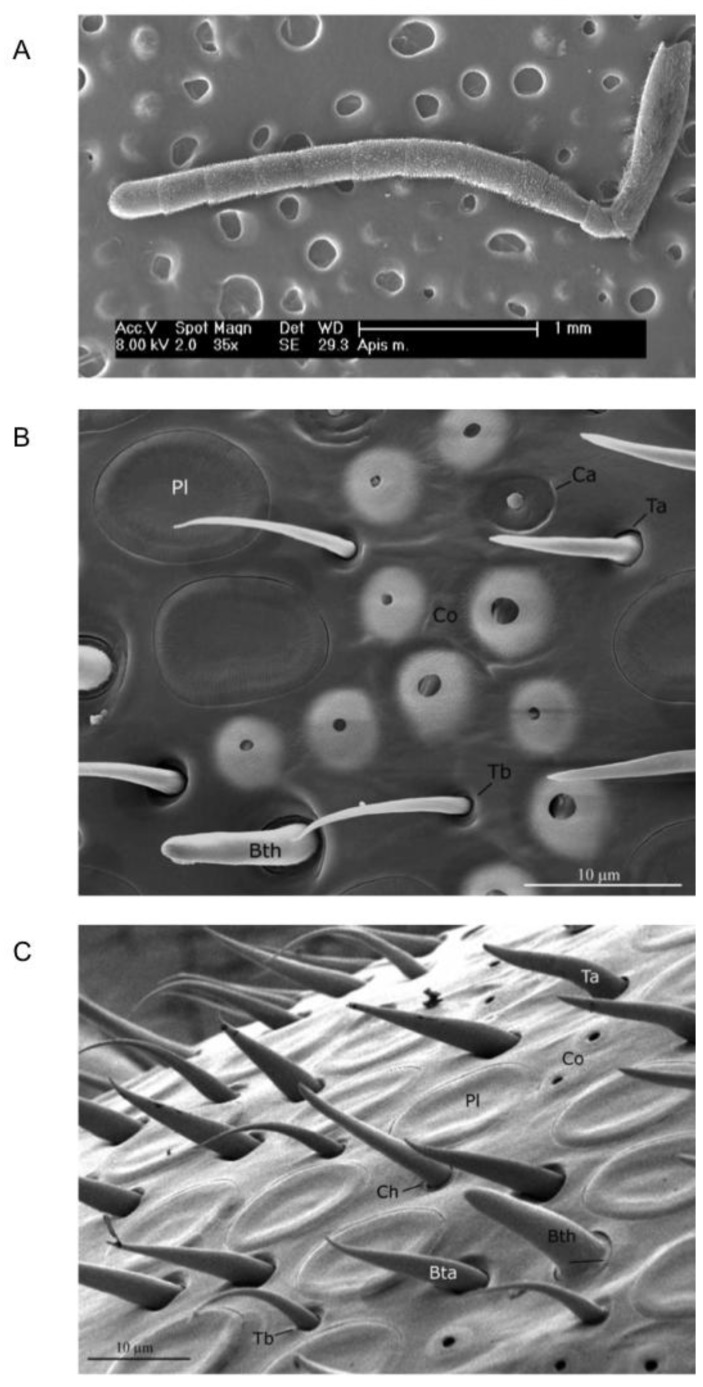
Scanning electron microscopy (SEM). (**A**) Low-magnification SEM image of the ventral view of *A. mellifera* left antenna. Segments from 3rd to 10th were imaged for subsequent counting of sensilla. (**B**) and (**C**) Scanning electron micrographs of the antenna of *A. mellifera* forager. Pl, sensillum placodeum; Ta, sensillum trichodeum type a; Tb, sensillum trichodeum type b; Ba, sensillum basiconicum thick; Bb, sensillum basiconicum tapered; Ch, sensillum chaeticum; Co, sensillum coeloconicum; Ca, sensillum campaniformium. (From [40]).

Frasnelli *et al.* [40] duplicated the behavioural results of Letzkus *et al.* [14] using forager Italian honeybees (*Apis mellifera ligustica* Spin.) and checked for morphological differences in the number of sensilla between the right and the left antenna of 14 honeybees. Both antennae of each bee were imaged from four different perspectives and all of the different types of sensilla were counted (Figure 4A). Results showed that putative olfactory sensilla (*placodea*, *trichodea*, *basiconica*—Figure 4B,C) were significantly more abundant on the right antennal surface than on the left antennal surface (mean difference of 3%), whereas sensilla not involved in olfaction (*campaniformia*, *coeloconica*, *chaetica*—Figure 4B,C) were more abundant on the left than on the right antennal surface (mean difference of 7%), but not on the tenth segment where no difference was observed. This opposing morphological asymmetry would suggest that behavioural asymmetries in other sensory domains might exist.

To complete the behavioural study conducted by Anfora *et al.* [20], an analysis of the number of olfactory sensilla present on the mason bees’ antennae has recently been conducted [41] and no significant difference in the number of the olfactory (and non-olfactory) sensilla between the right and the left antenna was observed.

In bumblebees, Anfora *et al.* [21] showed that only one class of olfactory sensilla, *trichodea type A*, is more abundant on the surface of the right antenna than on the left antenna. Although a slight tendency towards asymmetry was found for a second class, *i.e.*, *sensilla coeloconica*, this difference was not significant. Furthermore, *sensilla placodea*, the most common olfactory organs in Apoidea species, did not show any consistent asymmetrical distribution in *B. terrestris* [40].

An asymmetrical distribution of antennal sensilla has been shown in female wasps *Anastatus japonicus* Ashmead (Hymenoptera: Eupelmidae), an important egg parasitoid of several major insect pests [42]. Similar to the study by Letzkus *et al.* [14] and the one by Frasnelli *et al.* [40], the olfactory sensilla, *S. placodea*, were found to be more abundant on the right than left antenna, whereas the non-olfactory sensilla, *S. trichodea* and *S. basiconica*, were more abundant on the left than right antenna. Interestingly, *A. japonicus* is a non-social species and is not closely related to *A. mellifera*, but their antennal sensilla display a strong asymmetrical distribution pattern both quantitatively and spatially between the right and left antennae.

**Figure 5:**
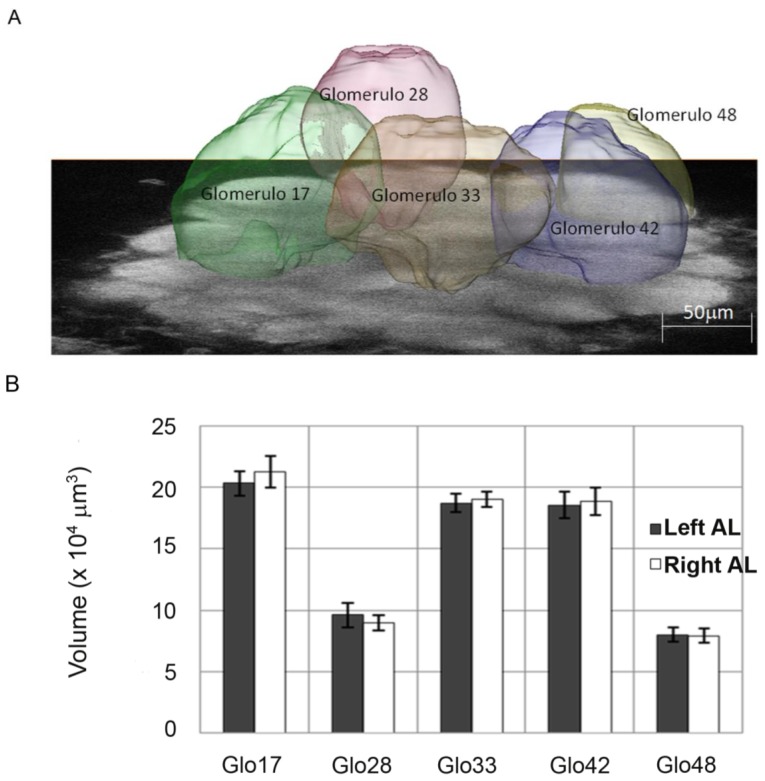
(**A**) Single image of the left antennal lobe of *Apis mellifera* acquired with two-photon microscopy at an imaging depth of approximately 80 µm. The reconstructed volume images of the analysed glomeruli are superimposed (From [18]). (**B**) Right and left absolute volumes of the 5 glomeruli of the honeybee antennal lobes that were investigated by Rigosi and colleagues [18]. Mean values are shown (±SEM, N = 12); (From [18]).

Although differences in number of sensilla and electrophysiological recordings possibly shed light on asymmetry in odour detection, they cannot *per se* account for the behavioural lateralizations that we have discussed, in particular the time-dependent shift of antenna access to memory. To address this issue in more depth, Rigosi *et al.* [18] investigated whether differences between the left and right sides were apparent at the central level, in the bee brain. From the antennae, odour receptor neurons send their information ipsilaterally to the first olfactory neuropil of the brain, the antennal lobes (ALs). Each AL is comprised of ~160 glomeruli, spherical structures that are invaded by one specific olfactory receptor class. Each odour elicits a specific spatio-temporal pattern of glomerular activation stimulated by action potentials from the olfactory receptor neurons. The glomeruli are linked by ~4000 local interneurons (LNs) that modulate the odour response pattern, which is then forwarded by ~800 projection neurons (PNs) to higher order brain areas (reviewed in [43]). Rigosi and colleagues [18] measured and compared the volumes of specific glomeruli in the left and right ALs (Figure 5). The volumetric measurements between sides were conducted in naïve foragers (not trained to PER for specific odours) to look for morphological correlates of peripheral asymmetry in ALs. The subset of glomeruli chosen for the analysis (Figure 5A) was the one that had been shown in previous studies to undergo plastic rearrangements of volume after odour experience [44] and to be involved in encoding information of those odours shown to trigger behavioural asymmetries with PER [17,20]. The AL imaging was performed using two-photon microscopy [45], which offers enhanced penetration depth and a higher axial resolution than conventional fluorescence microscopy. This improved the spatial resolution with respect to the only work published previously comparing AL morphology in the two sides of the brain [46]. With this technique Rigosi and colleagues [18] could compare data of the left and right side directly within each bee and they showed that the chosen subset of glomeruli did not differ in volume between sides (Figure 5B). However, although no differences were revealed at the level of glomerular volumes, other morphological rearrangements within the AL network might take place without glomerular volume being affected (e.g., number of synapses, dendritic branches, *etc.*). In addition, differences between sides might be present at the functional level (the odour-dependent patterns of activity in the AL network). A first study on the AL response pattern did not reveal any asymmetries [47], but the limited contrast of the functional signals could have masked small effects. Two-photon microscopy might overcome this problem as it has been shown to increase resolution as well as signal strength also in functional imaging of the honeybee brain [48].

## 5. Conclusions

Since it has been hypothesized (for vertebrates) that lateralization at the population level may emerge as a result of social selective pressures [33], it could be that the pattern observed in these species of Hymenoptera Apidae is due to the different degrees of sociality of the species.

Overall the various studies of laterality in bees show that social bees are lateralized at the population level in tasks of odour association with a food reward. Despite variations in the exact methods used in conditioned learning and memory recall, there is strong evidence for predominance of the right antenna in recall of short-term olfactory memory. The evidence that it is the left antenna used in retrieval of long-term memory suggests that memory storage uses, and may be enhanced by, lateralized processes. Given that non-social mason bees are lateralized at the individual and not population level, it is possible that such lateralized processes are also important in this species albeit without the same direction of bias in most individuals. Modified methods of testing recall will be needed to confirm this.

Population biases are present in social interactions in honeybees and here it is use of the right antenna that leads to appropriate behaviour between bees from the same colony and from different colonies. Considering the role of the right antenna in learning and short-term memory recall of odours, this may indicate that communication between bees relies on short-term memory and may depend on memories that are flexible and continually up-dated in social interactions.

In honeybees the olfactory receptor neurons in the right antenna show higher electrophysiological responses compared to those in the left antenna [20]. This antennal asymmetry is partially explained by a difference in the number of the olfactory sensilla between the right and the left antenna, the right antenna having more olfactory sensilla [40]. However, such a small difference (3%–7%) in the number of olfactory sensilla might not entirely explain the superior performance of the right antenna in learning or recall and also the one order of magnitude (18%–20%) greater difference observed in the electrophysiological responses.

No asymmetries have been so far found at the level of the antennal lobes, possibly due to a lack of control of bees’ previous odour exposure and experience. Further studies are currently in progress and are looking at the coding of odours in the antennal lobes.

Bees with only their right antenna in use are able to associate an odour with a food reward and recall the short-term memory of this task. This lateralization could be, at least partially, explained by the expression of NLG1, one of the proteins found on synaptic membranes of neurons and producing a trans-synaptic bridge that facilitates maturation and specification of synapses. The level of NLG1 expression in bees with only their right antenna in use is slightly lower than in bees with both antennae intact, whereas bees with only their left antenna intact have a much lower expression level of NLG1. Further studies on the levels of the proteins involved in memory formation may also shed light on the lateralized processes involved in encoding of memory in bees.

Finally, we would like to emphasise that there is now a considerable amount of evidence demonstrating that the honeybee’s behaviour is lateralized. This fact should be taken into account in future research on the nervous system and behaviour of honeybees. So far the honeybee has provided the most convincing evidence that lateralization is not limited to vertebrate species. It appears that even a small brain benefits from breaking symmetry along the left-right axis.
